# Inhibition of the lectin pathway of complement ameliorates hypocomplementemia and restores serum bactericidal activity in patients with severe COVID‐19

**DOI:** 10.1002/ctm2.980

**Published:** 2022-07-15

**Authors:** Nicholas J. Lynch, Andrew C. Y. Chan, Youssif M. Ali, Priyanka Khatri, Ifeoluwa E. Bamigbola, Gregory Demopulos, Muriel Paganessi, Alessandro Rambaldi, Wilhelm J. Schwaeble

**Affiliations:** ^1^ Department of Veterinary Medicine University of Cambridge Cambridge UK; ^2^ Omeros Corporation Seattle Washington USA; ^3^ Unit of Hematology Azienda Socio‐Sanitaria Territoriale Papa Giovanni XXIII Bergamo Italy; ^4^ Department of Oncology‐Hematology University of Milan Milan Italy; ^5^ Department of Microbiology and Immunology, Faculty of Pharmacy Mansoura University Mansoura Egypt

Dear Editor,

Complement activation is a key mediator of thrombosis, inflammation and tissue damage during acute SARS‐CoV‐2 infection. Complement components are depleted during acute infection, and the proinflammatory anaphylatoxins C3a and C5a, which play a major role in the development of acute respiratory distress syndrome (ARDS), are released.^1^, ^2^, ^3^, ^4^ Extensive complement activation may be prognostic of poor outcome – higher degrees of activation, especially of C3, being associated with fatality.^4^ Complement inhibitors are therefore considered promising therapeutics for tackling acute COVID‐19 ARDS; potential targets include C3 and the C5a/C5aR axis as well as the lectin and terminal pathways.^5^, ^6^

Analysis of post‐mortem tissue from patients who succumbed to COVID‐19 revealed severe endothelial damage characteristic of microangiopathies, with deposits of complement activation products, most notably the lectin pathway serine protease MASP‐2.^7^, ^8^ We have previously found evidence of a novel mechanism of lectin pathway activation, driven by a direct interaction between the SARS‐CoV‐2 nucleocapsid protein and the lectin pathway serine protease MASP‐2.^9^

Narsoplimab is a fully humanised monoclonal antibody that targets MASP‐2, blocking the activation of the lectin pathway (S1). In March 2020, we conducted a small clinical study using narsoplimab to treat six patients with severe COVID‐19. All narsoplimab‐treated patients recovered, survived and were discharged from the hospital. Recovery correlated with rapid and sustained reduction of circulating endothelial cells (a marker of endothelial damage) and an improvement in serum levels of interleukin‐6 (IL‐6), IL‐8, C‐reactive protein, lactate dehydrogenase and aspartate aminotransferase. d‐Dimer levels, a marker of thrombosis, were also normalised. Two retrospective control groups with similar entry criteria and baseline characteristics showed mortality rates of 32% and 53%. No adverse drug reactions were reported in the study.^6^

Here we report on a follow‐on study, conducted later in the pandemic, which specifically addresses the impact of narsoplimab on complement activity in COVID‐19.

We analysed longitudinal plasma samples from nine severely ill COVID‐19 patients treated with narsoplimab and nine untreated controls, all admitted to the ICU of Papa Giovanni XXIII Hospital in Bergamo, Italy during the fourth quarter of 2020. All patients were PCR positive for SARS‐CoV‐2 and had ARDS according to the Berlin criteria, requiring mechanical ventilation. The most severely ill patients were chosen for treatment with narsoplimab, all of whom had failed other therapies and had multiple comorbidities. Two patients selected for narsoplimab treatment died early on, the first of complications of pre‐existing cardiomyopathy early after narsoplimab initiation and the second, for whom narsoplimab was initiated 13 days following intubation, of multi‐organ failure.

All patients received enoxaparin, dexamethasone and 500‐mg azithromycin daily. Patients with active systemic bacterial or fungal infections requiring further antimicrobial therapy were not eligible for narsoplimab treatment. In the treated cohort, narsoplimab (4 mg/kg) was administered intravenously twice weekly for 2–4 weeks. Normal control plasma samples were collected from 17 healthcare worker volunteers seronegative for SARS‐CoV‐2. The characteristics of the narsoplimab‐treated and untreated control patients with COVID‐19, together with laboratory findings on admission, are shown in Table 1.

**TABLE 1:** Clinical parameters for the subjects included in this study prior to treatment

Clinical characteristics	Narsoplimab‐treated COVID‐19 cohort (*N* = 9)		Untreated COVID‐19 cohort (*N* = 9)	
	Median	Range	Median	Range
Age – years	62	41–79	74	50–79
Weight – kg	90	75–105	85	65–130
BMI – kg/m^2^	27.8	25.5–32.5	29	21–55
Sex				
Female	1/9		3/9	
ARDS severity (Berlin criteria)				
Mild			2/9	
Moderate	3/9		5/9	
Severe	6/9		2/9	
Laboratory findings	Median	Range	Median	Range
PaO_2_:FiO_2_ ratio	140	110–250	180	80–284
White cell count – per mm^3^	8470	4600–21 520	7100	3140–14 590
Lymphocyte count – per mm^3^	550	350–1940	390	142–750
Platelet count – ×10^3^ per mm^3^	247	154–313	223	102–444
Haemoglobin – g/dl	12.7	11.3–15.4	10.3	7.9–14.8
Other findings (reference ranges)				
C‐reactive protein (.0–1.0 mg/dl)	14.5	1.9–17.9	9.3	.3–26.9
Lactate dehydrogenase (120/246 U/L)	443	312–582	346.5	190–494
Aspartate aminotransferase (13–40 U/L)	55	19–89	40	10–59
Alanine aminotransferase (7–40 U/L)	44	22–252	23	11–148
Creatinine (.3–1.3 mg/dl)	.79	.48–1.13	1.85	.81–4.34
d‐Dimer (<500 ng/ml)	1141	354–4471	1730	288–5020
Comorbidities				
Diabetes	2/9		3/9	
Hypertension	5/9		6/9	
Dyslipidaemia	1/9		3/9	
Cardiovascular disease	0/9		5/9	
Obesity (BMI ≥ 30 kg/m^2^)	2/8^a^		4/9	
Overweight (BMI ≥ 25 kg/m^2^)	6/8^a^		8/9	
Radiologic findings				
Bilateral interstitial abnormalities	9/9		9/9	

*Note*: Data collected on day 0 of the study, immediately prior to administration of the first dose of narsoplimab in the treated cohort. The most severely ill patients, as judged by the Berlin criteria, were assigned to the treatment arm; an equal number of patients being treated in the same ICU were assigned to the control arm. Seventeen seronegative healthcare workers were recruited as negative controls, matched as closely as possible to the patient cohorts (4 out of 17 females; median age 59, range 51–66; median BMI 28, range 22–44).

Abbreviation: ARDS, acute respiratory distress syndrome.

^a^ Data missing for one patient.

On admission, all 18 COVID‐19 patients showed hypocomplementemia, that is low CH_50_ values (Figure 1A), and elevated plasma C5a (Figure 1B), with no significant differences between the treated and untreated groups, despite the untreated patients having less severe disease than those chosen for narsoplimab treatment (Table 1). The CH_50_ is an end–end measurement of haemolytic activity driven by the classical pathway, but is affected by the consumption of components that are common to all three pathways, and, therefore, does not distinguish which pathway is activated: Likewise, the anaphylatoxin C5a is produced by all three pathways. Treatment with narsoplimab resulted in an immediate improvement of complement haemolytic activity (CH_50_) and a reduction in C5a. In the untreated group, C5a remained elevated, and CH_50_ values low, for the first 9 days of the study, the CH_50_ beginning to normalise shortly before discharge.

**FIGURE 1:**
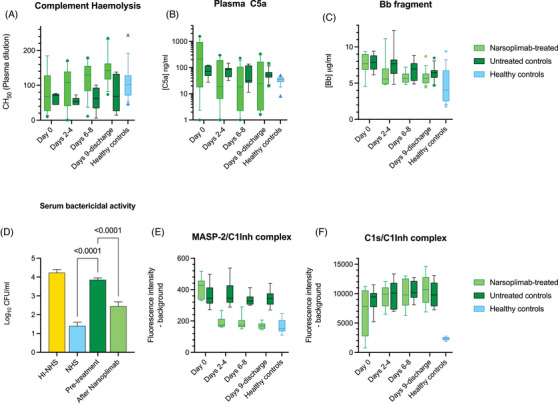
Narsoplimab treatment ameliorates hypocomplementemia in acute COVID‐19. Prior to treatment, the study subjects had significantly impaired complement‐mediated haemolysis (panel A, *p* < .001 vs. healthy controls, the Supplementary Method, S3 section). Levels of C5a were elevated (panel B; *p* = .032, the Supplementary Method, S4 section). In the narsoplimab‐treated group, both CH_50_ and C5a had returned to normal 3 days after the first dose and remained normal for the duration of the study. In the untreated group, CH_50_ values were significantly lower (*p* = .0019), and C5a levels significantly higher (*p* = .020), than in the treated group throughout the remainder of the study. Soluble Bb, a marker of alternative pathway activation, was high on admission and stayed high throughout the study but was significantly reduced in the narsoplimab‐treated group (*p* = .027 vs. untreated controls; panel C, the Supplementary Method, S4 section). The serum bactericidal activity against *Klebsiella pneumoniae* was compromised by hypocomplementemia on admission but restored by treatment with narsoplimab (panel D; NHS = normal human serum, HI‐NHS = heat‐inactivated NHS; post‐treatment samples taken 6–8 days after first treatment, the Supplementary Method, S5 section). At the start of the study, all patients had high levels of MASP‐2/C1Inh and C1s/C1Inh in their plasma, indicative of lectin pathway and classical pathway activation, respectively. Narsoplimab treatment reduced MASP‐2/C1Inh to levels seen in healthy control plasma directly after the first dose (panel E, the Supplementary Method, S6 section). Plasma MASP‐2/C1Inh levels in the control group of patients with severe acute COVID‐19 that did not receive narsoplimab were significantly (*p* < .001) higher than in the treated group for the rest of the study. In contrast, narsoplimab had no effect on the classical pathway‐driven production of C1s/C1Inh, which remained high in both patient groups throughout the study (F). All results are means of duplicates. *N* = 9 per patient group; 17 for seronegative healthy controls. Results were analysed using two‐way ANOVA with Dunnett's correction for multiple comparisons.

The alternative pathway marker factor Bb was elevated on admission, up to approximately 8 μg/ml, and did not return to normal in either the treated or untreated patient groups. However, Bb levels dropped significantly after narsoplimab treatment (1C), suggesting that the lectin pathway plays a significant role in driving alternative pathway activation during acute COVID‐19.

In a separate study of secondary infection in hospitalised COVID‐19 patients,^10^ we demonstrated that serum bactericidal activity (SBA) against *Klebsiella pneumoniae* is compromised in plasma from acute COVID‐19 patients. This held true in the patients studied here. SBA was significantly restored after treatment with narsoplimab (1D).

Neither the production of C5a nor a drop in CH_50_ reveals which complement pathway is activated in acute COVID‐19, as these parameters are affected by the activation of all three pathways. To address this limitation, we used a novel bead‐based fluorescent immunoassay to measure the formation of C1s/C1 inhibitor (C1Inh) and MASP‐2/C1Inh complexes, specific markers of classical pathway and lectin pathway activation, respectively (S6). The formation of these complexes is upstream to the convergence of the complement pathways, and they therefore provide ideal markers of lectin pathway and classical pathway activation.

In the narsolimab‐treated cohort, MASP‐2/C1Inh complexes dropped back below the levels seen in the healthy controls after the first administration of narsoplimab and remained below healthy control levels seen throughout the duration of the study, whereas, in the untreated group, MASP‐2/C1Inh complexes were consistently high throughout the study and significantly higher than in the control group (1E). In contrast, narsoplimab had no direct effect on the plasma levels of C1s/C1inh complexes, which remained elevated in both patient groups throughout the study (1F). The level of C1s/C1inh broadly correlated with anti‐SARS‐CoV‐2 antibody levels. Most patients were admitted with low levels of IgM and low‐to‐moderate levels of IgG, the latter increasing during the hospital stay (Figure S2). Despite this maturation of the antibody response, treatment with narsoplimab was sufficient to suppress C5a production and restore haemolytic activity and SBA against *K. pneumoniae*.

In conclusion, targeting the lectin pathway may suffice to reduce anaphylatoxin release below the threshold for maintaining ARDS and restore the bactericidal activity and opsonisation required for defence against secondary infection. This approach might be preferable to targeting C3, which would render the patient susceptible to opportunistic infections, or to plasma replacement therapy, which would restore complement‐mediated SBA, but might exacerbate ARDS.

## FUNDING INFORMATION

This work was supported by the NIHR/UKRI Grant no. COV0170 Humoral Immune Correlates of COVID‐19 (HICC) and by the Fondazione Cariplo grant Bio‐Banking of COVID‐19 Patient Samples to Support National and International Research (Covid‐Bank).

## CONFLICT OF INTEREST

WS, NL, YA and AR are consultants to Omeros Corporation which is developing inhibitors of the lectin pathway. GD is employed by Omeros Corporation. The other authors have no conflicts of interest to declare.

## Supporting information



Supporting Information
