# Supplementary material for: Inhibition of the lectin pathway of complement ameliorates hypocomplementemia and restores serum bactericidal activity in patients with severe COVID‐19
Source: Clin Transl Med. 2022 Jul 15;12(7):e980. doi: 10.1002/ctm2.980 (PMC9286524; doi:10.1002/ctm2.980)
Supplement: Supplementary file 1 — Supporting Information [file CTM2-12-e980-s001.docx]

**Supplementary Information**

**Supplementary Background**

*S1: Complement Activation Pathways and Narsoplimab Mechanism of Action*


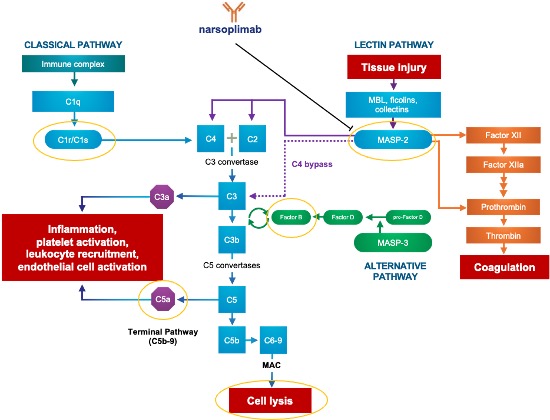


**Figure S1**. Schematic illustrating the complement system, aspects of its interaction with the coagulation system, and the mode of action of narsoplimab. Narsoplimab is an inhibitor of MASP-2, a key enzyme in the lectin pathway that cleaves C4 into C4b and C4a, and then C4b bound C2 (C4b2) into the C3 convertase C4b2a, thus inhibiting lectin pathway-driven C3 conversion. Secondary substrates include prothrombin and, likely, factor XII. Thus narsoplimab interferes with secondary haemostasis though both the intrinsic and extrinsic pathways. Markers of complement activation that were followed in this study are circled in orange. Adapted from Rambaldi et al., 2020 (1).

**Supplementary Data**

*S2: Antibody response in severe acute COVID-19.*

**Figure S2.** Plasma samples from the treated and untreated patient groups (n=9 per group) were assayed for anti-SARS-CoV-2 nucleocapsid protein, using a Luminex-based system, essentially as described previously (2). IgM levels were slightly elevated on admission to the ICU in comparison to healthy (seronegative) controls (n=17) and did not change significantly during the hospital stay, whereas IgG levels were elevated on admission and increased significantly during first four days (p=0.022 for the time effect; 2-way ANOVA with Dunnett’s correction). Narsoplimab had no significant effect on antibody levels.

**Supplementary Methods**

***S3: Measurement of complement haemolysis (CH_50_)***

Antibody-driven complement lysis of sheep erythrocytes (SE) was measured using rabbit anti-sheep IgG coated SE, as described by Costabile at al. (3). Sheep erythrocytes (Oxoid) were washed 3 times using GVB buffer (10mM barbital, 145mM NaCl, 0.1%w/v gelatine), containing 10mM EDTA. The final concentration of RBCs was adjusted to 1×10^9^/mL. RBCs were sensitized by incubation with anti-sheep RBCs (Sigma S1389, diluted 1:200) at 37°C with gentle shaking for 30 minutes. Finally, RBCs were washed with GVB buffer containing 2mM Ca^2+^ and 1mM Mg^2+^ (GVB^++^). Plasma samples were serially diluted in 100µL GVB^++^ buffer in 96-well plates, and 10^7^ RBCs in an equal volume of GVB^++^ were added to each well. Wells containing buffer only were used as a negative control. Wells containing water instead of buffer/plasma were used as a positive control (nominally 100% lysis). After 30 minutes of incubation at 37°C, plates were centrifuged, 100 µL of the supernatant were aspirated and released haemoglobin determined by measurement of the OD at 405 nm. The percentage of haemolysis was calculated and plotted against the plasma dilution to determine the CH_50_.

***S4: C5a and Factor Bb ELISAs***

Circulating C5a was measured using a proprietary sandwich ELISA supplied by R&D systems (Cat. No. DY2037). The factor B cleavage product Bb, a marker of alternative pathway activation, was determined using the MicroVue factor B kit (Quidel).

***S5: Serum bactericidal assay (SBA)***

*K. pneumoniae* isolates were grown in nutrient broth at 37°C for overnight with gentle shaking. Next day, 10 mL of fresh nutrient broth were seeded with 100 μL of overnight bacterial culture and incubated at 37°C with gentle shaking until mid-logarithmic phase. Bacterial cultures were collected, washed twice using BBS (4mM barbital, 145mM NaCl, 2mM CaCl2, 1mM MgCl_2_, pH 7.4) and then adjusted to a final concentration of 1×10^7^ CFU mL−1. 1×10^5^ CFU were incubated with 50% serum from HCW or sera from acute/convalescent COVID-19 patients in BBS at 37°C with gentle shaking. After 2 hours, samples were taken and plated out on a nutrient agar plate for overnight at 37°C. Serum bactericidal activity was calculated by measuring the decrease in the viable bacterial count recovered after 2-hour incubation with each serum compared to heat-inactivated normal human serum (HI-NHS).

***S6: Luminex assay for MASP-2/C1Inh and C1s/C1Inh complexes***

***Background***: An inherent problem with assays that measure complement depletion or complement activation products is that they fail to distinguish which pathway is activated, because the majority of downstream complement components are common to all three pathways (fig. S1). For example, in the CH_50_ assay described here, antibody-sensitized sheep erythrocytes activate the classical pathway leading to lysis of the erythrocytes by the terminal pathway membrane attack complex (MAC). C4, C2 and all downstream components are common to both the classical and lectin pathways, while C3 and all subsequent components are common to all three pathways. Thus, the CH_50_, nominally a measure of classical pathway activation, is affected by activation of any of the pathways. Likewise, measurements of C3b deposition or C5b-9 formation are not pathway-specific, nor are measurements of anaphylatoxin release.

This problem can be circumvented by following the production of complexes between the classical or lectin pathway serine proteases and their natural inhibitor C1Inh (aka SERPIN G1 or C1-INH). These complexes are formed upstream of the components that are common to both pathways and, therefore, represent unique markers of either classical or lectin pathway activation. The serine proteases are found in plasma as zymogens, loosely bound to C1Inh.

MASP-2 is associated with one of several lectin pathway pattern recognition molecules. When sufficient lectin pathway-specific recognition molecules bind in close proximity on an activating surface, zymogen MASP-2 is cleaved into two disulphide-linked chains, either by another molecule of MASP-2 or by MASP-1. Cleaved MASP-2 is the active form of the enzyme, which cuts its substrates, C4 and C2. MASP-2 is then inactivated by C1Inh, which functions as a pseudo-substrate and binds covalently to activated MASP-2, forming a stable 1:1 complex.

Similarly, the classical pathway recognition complex C1 is a multimolecular complex comprising the recognition component C1q and heterodimers of the zymogen forms of C1r and C1s, loosely bound to C1Inh. When C1 is activated (typically by binding to immune complexes), C1r and C1s activate sequentially and are subsequently inactivated by C1Inh, yielding covalent complexes of serine proteases with C1Inh.

Thus, the levels of MASP-2/C1Inh and C1s/C1Inh in a sample of plasma or serum provide a clear measure of recent lectin pathway or classical pathway activation, respectively.

Kajdacsi *et al.* described sandwich ELISAs to measure complexes of complement serine proteases with C1Inh (4). They used a rat anti-human MASP-2 monoclonal from Hycult Biotech (mAb 8B5) as a capture antibody to measure MASP-2/C1-INH complexes in healthy humans and in hereditary angioedema (HAE) patients. Hansen *et al.* used the same capture antibody in an ELISA assay to measure MASP-2/C1-INH complexes in HAE patients (5). Both groups reported that MASP-2/C1-INH complexes were only detected using high serum concentrations (10-20%).

To develop sensitive assays suitable for screening individual patient samples at serum concentrations less than 10% for the presence and/or amount of MASP-2/C1Inh complex or C1s/C1Inh, we transferred the sandwich assays to a bead-based fluorescent format and multiplexed them, using the Luminex platform.

***Antibody-coated magnetic beads***: A panel of monoclonal antibodies known to bind to MASP-2 and C1s were tested as capture antibodies. Antibodies were diluted to 50µg/ml in phosphate buffered saline (PBS) and immobilized by carbodiimide coupling on MagPlex magnetic polystyrene microspheres (Luminex), using methods originally described in the Luminex (xMAP) Cookbook (4th Edition) and Xiong *et al.* (6).

1.25x10^7^ superparamagnetic MagPlex beads were activated with 1-ethyl-3-(3-dimethylaminopropyl) carbodiimide hydrochloride (Thermo Fisher Scientific) in the presence of N-hydroxysuccinimide (Thermo Fisher Scientific) in 100 mM MES buffer, pH 6.0. After washing with Dulbecco’s phosphate-buffered saline (DPBS), pH7.4 (without calcium and without magnesium), 50µg of capture antibody was incubated with the beads in DPBS, pH 7.4, for 2 hours on a rotator at room temperature. After coupling, any remaining reactive sites on the beads were blocked by incubation with PBS containing 0.05% TWEEN 20, pH 7.4 (PBS TBN). Coupled beads were then washed with DPBS, pH7.4 and stored in storage buffer (DPBS, 1% BSA, and 0.05% w/v NaN3). BSA-coated beads were prepared as negative controls. MagPlex beads with different emission spectra were used for anti-C1s, anti-MASP-2 and BSA coated beads, to allow the assay to be multiplexed.

***Assay procedure***: Antibody- and BSA-coupled MagPlex beads were diluted in assay buffer (DPBS, 1% BSA, 0.8% Poly Vinyl Pyrrolidone, 0.5% Poly Vinyl Alcohol, 0.05% w/v NaN_3_) to a final concentration of 30 microspheres/μL of each type of bead. Fifty μL of this mixture was aliquoted into each well of a 96-well plate. An equal volume of plasma or serum diluted in assay buffer was added to the wells, mixed and incubated for 1hr at 37°C on a shaker. The beads were washed 3 times with PBST (PBS and 0.05% v/v Tween-20) by retaining them with a magnetic separator, aspirating the supernatant and adding 100μL of fresh wash buffer. After washing, the beads were resuspended in 50μL of assay buffer and bound ligand was detected by adding a biotinylated anti-C1Inh polyclonal antibody (R&D Systems, BAF2488) diluted 1:1000 in assay buffer. The beads were incubated with the detection antibody at 37°C on a shaker for 30min, before being washed 3x as described above. Streptavidin R-phycoerythrin (SAPE; Thermo Fisher Scientific) was diluted to 1μg/mL in assay buffer, 100μl added to each well, mixed and incubated for 37°C on a shaker. After washing as above, the beads were resuspended in 100μl of wash buffer and analyzed on a Bio-Rad Bio-Plex 200 with the “High RF1 Target” setting on. The data was captured with the Bio-Plex Manager Software. The data is reported as Fluorescent Intensity (FI) – background, with background being a no-serum/plasma control.

***Antibody selection***: In preliminary experiments designed to test pairs of capture and detection antibodies, we prepared control sera for the MASP-2/C1Inh assay by incubating normal human serum with mannan-agarose, artificially activating the LP and releasing MASP-2/C1inh into the sample. Likewise, a positive control for C1s/C1Inh complexes was prepared by incubating normal human serum with sheep anti-HSA, generating immune complexes in situ to artificially activate the CP and release C1s/C1inh into the sample. Serial dilutions of these sera, ranging from 1:10 to 1:1280 were assayed as described above. Controls were: Non-activated NHS, BSA-coated beads, no-serum (buffer only) reactions, and mixtures omitting the detection antibody. The following mAb were shown to work well as capture Ab.

• Anti-MASP-2 humanized mouse mAb, C1NA0, Omeros Corporation

• Anti-C1s affinity-purified polyclonal, Proteintech (14554-1-AP)

These capture antibodies gave a straightforward log/linear relationship between sample concentration and fluorescent intensity at sample dilutions from 1:10 to 1:640, with the signal falling to background levels at 1:1280. The anti-MASP-2 mAb clone 8B5 (Hycult Biotech), previously used successfully in sandwich ELISAs, performed poorly in the Luminex assay, with poor sensitivity and a low signal-to-noise ratio. A full list of the antibody pairs tested is available from the corresponding author on request.

**Assay performance**: Acute COVID-19 infection is known to lead to complement activation, de-complementation and the release of complement activation products, e.g., C3a and C5a. We tested the bead-based C1Inh complex assays using plasma from 40 patients with severe acute COVID-19, who were recruited at the Royal Papworth Hospital, UK. The WHO clinical scores for the patients ranged from 3-7, and 19 of them required extracorporeal membrane oxygenation (ECMO). Nineteen of the patients survived; 21 succumbed to the disease. Thirty uninfected health care workers (HCW) served as controls. Serial dilutions of pooled acute phase plasma were used for standards (range 1:10-1:1280). Individual samples were diluted 1:50 in assay buffer. A high and low standard (pooled acute and NHS) were included at 3 separate locations on each plate to determine intra- and inter-plate variation. The standard curves for both assays were straight log/linear relationships, with usable plasma dilutions ranging from 1:20 to 1:640 (fig. S3). The absolute fluorescence signal for the C1s/C1Inh complex is approximately 10-fold higher than that for the MASP-2/C1Inh complex, perhaps reflecting the difference in serum concentration between MASP-2 and C1s.

**Figure S3**. Examples of standard curves for the MASP-2/C1Inh and C1s/C1Inh assays. Plasma was taken from 40 severe acute COVID-19 patients, 7 days after admission to the ICU, and pooled. Serial dilutions were prepared and assayed using a mixture of MagPlex beads coated with anti-MASP-2 and anti-C1s antibodies. Beads coated with BSA served as a negative control. Results are means of duplicates and representative of >5 experiments.

MASP-2/C1Inh complexes and C1s/C1Inh complexes were significantly elevated in all of the hospitalized acute COVID-19 patients compared to the healthy controls, indicating activation of both the LP and the CP. (Fig. S4)

Inter-assay and intra-assay variability were strikingly low (CV=4% and 5%, respectively), *provided* that the same preparations of antibody coated beads were used. However, there was significant variation between different bead preparations, presumably reflecting differences in coupling efficiency between preparations. For this reason, we chose to use a single preparation of antibody-coupled beads for all of the assays described in the main text of this manuscript.

For larger studies, requiring more than one set of coupling reactions, it should be possible to normalize the fluorescence values to a standard set of beads, provided that appropriate standards are included on each plate.

An alternative method of standardization, in which standards and positive controls are prepared by mixing recombinant C1Inh and either recombinant C1s or recombinant MASP-2 in stochiometric amounts, and purifying the resulting C1s/C1Inh or MASP-2/C1Inh complexes by size exclusion chromatography is in development. This method has previously been using to standardize sandwich ELISAs (4), and has the advantage of permitting comparisons between results obtained in different laboratories.

**Figure S4**. MASP-2/C1Inh complexes are elevated in acute COVID-19. Plasma was taken from 40 severe acute COVID-19 patients, 7 days after admission to the ICU, and 30 seronegative healthcare workers. Samples (diluted 1:50) were assayed using the Luminex assay described in this supplement.

***Supplementary References***

1. Rambaldi A, Gritti G, Micò MC, Frigeni M, Borleri G, Salvi A, Landi F, Pavoni C, Sonzogni A, Gianatti A, Binda F, Fagiuoli S, Di Marco F, Lorini L, Remuzzi G, Whitaker S, Demopulos G. Endothelial injury and thrombotic microangiopathy in COVID-19: Treatment with the lectin-pathway inhibitor narsoplimab. Immunobiology. 2020 Nov;225(6):152001. doi: 10.1016/j.imbio.2020.152001. Epub 2020 Aug 9. PMID: 32943233; PMCID: PMC7415163.
2. Baxendale HE, Wells D, Gronlund J, Nadesalingham A, Paloniemi M, Carnell G, Tonks P, Ceron-Gutierrez L, Ebrahimi S, Sayer A, Briggs JAG, Ziong X, Nathan JA, Grice G, James LC, Luptak J, Pai S, Heeney JL, Lear S, Doffinger R. Critical Care Workers Have Lower Seroprevalence of SARS-CoV-2 IgG Compared with Non-patient Facing Staff in First Wave of COVID19. J Crit Care Med (Targu Mures). 2021 Aug 5;7(3):199-210. doi: 10.2478/jccm-2021-0018. PMID: 34722923; PMCID: PMC8519390.
3. Costabile M. Measuring the 50% haemolytic complement (CH50) activity of serum. J Vis Exp. 2010 Mar 29;(37):1923. doi: 10.3791/1923. PMID: 20351687; PMCID: PMC3168207.
4. Kajdácsi E, Jandrasics Z, Veszeli N, Makó V, Koncz A, Gulyás D, Köhalmi KV, Temesszentandrási G, Cervenak L, Gál P, Dobó J, de Maat S, Maas C, Farkas H, Varga L. Patterns of C1-Inhibitor/Plasma Serine Protease Complexes in Healthy Humans and in Hereditary Angioedema Patients. Front Immunol. 2020 May 5;11:794. doi: 10.3389/fimmu.2020.00794. PMID: 32431708; PMCID: PMC7214733.
5. Hansen CB, Csuka D, Munthe-Fog L, Varga L, Farkas H, Hansen KM, Koch C, Skjødt K, Garred P, Skjoedt MO. The Levels of the Lectin Pathway Serine Protease MASP-1 and Its Complex Formation with C1 Inhibitor Are Linked to the Severity of Hereditary Angioedema. J Immunol. 2015 Oct 15;195(8):3596-604. doi: 10.4049/jimmunol.1402838. Epub 2015 Sep 14. PMID: 26371246.
6. Xiong X, Qu K, Ciazynska KA, Hosmillo M, Carter AP, Ebrahimi S, Ke Z, Scheres SHW, Bergamaschi L, Grice GL, Zhang Y; CITIID-NIHR COVID-19 BioResource Collaboration, Nathan JA, Baker S, James LC, Baxendale HE, Goodfellow I, Doffinger R, Briggs JAG. A thermostable, closed SARS-CoV-2 spike protein trimer. Nat Struct Mol Biol. 2020 Oct;27(10):934-941. doi: 10.1038/s41594-020-0478-5. Epub 2020 Jul 31. PMID: 32737467; PMCID: PMC7116388.
